# Solid Lipid Nanoparticle Carrier Platform Containing Synthetic TLR4 Agonist Mediates Non-Viral DNA Vaccine Delivery

**DOI:** 10.3390/vaccines8030551

**Published:** 2020-09-21

**Authors:** Jasmine E. Francis, Ivana Skakic, Chaitali Dekiwadia, Ravi Shukla, Aya C. Taki, Anna Walduck, Peter M. Smooker

**Affiliations:** 1 School of Science, RMIT University, 264 Plenty Road, Bundoora, VIC 3083, Australia; s3476832@student.rmit.edu.au (J.E.F.); ivana.skakic@rmit.edu.au (I.S.); anna.walduck@rmit.edu.au (A.W.); 2 RMIT Microscopy & Microanalysis Facility, School of Science, RMIT University, Melbourne, VIC 3001, Australia; chaitali.dekiwadia@rmit.edu.au; 3 NanoBiotechnology Research Laboratory, School of Science, RMIT University, Melbourne, VIC 3001, Australia; ravi.shukla@rmit.edu.au; 4 Department of Veterinary Biosciences, Melbourne Veterinary School, Faculty of Veterinary and Agricultural Sciences, The University of Melbourne, Parkville, VIC 3010, Australia; aya.taki@unimelb.edu.au

**Keywords:** DNA vaccine, solid lipid nanoparticle, nanobiotechnology, *Helicobacter pylori*, lipoplex

## Abstract

There is a growing demand for better delivery systems to improve the stability and efficacy of DNA vaccines. Here we report the synthesis of a non-viral DNA vaccine delivery system using a novel adjuvanted solid lipid nanoparticle (SLN-A) platform as a carrier for a DNA vaccine candidate encoding the Urease alpha (UreA) antigen from *Helicobacter pylori*. Cationic SLN-A particles containing monophosphoryl lipid A (adjuvant) were synthesised by a modified solvent-emulsification method and were investigated for their morphology, zeta potential and in vitro transfection capacity. Particles were found to bind plasmid DNA to form lipoplexes, which were characterised by electron microscopy, dynamic light scattering and fluorescence microscopy. Cellular uptake studies confirmed particle uptake within 3 h, and intracellular localisation within endosomal compartments. In vitro studies further confirmed the ability of SLN-A particles to stimulate expression of pro-inflammatory cytokine tumor necrosis factor alpha (TNF-α) in human macrophage-like Tohoku Hospital Pediatrics-1 (THP-1) cells. Lipoplexes were found to be biocompatible and could be efficiently transfected in murine immune cells for expression of recombinant *H. pylori* antigen Urease A, demonstrating their potential as a DNA vaccine delivery system.

## 1. Introduction

Compared to traditional approaches to vaccination, DNA vaccines offer a safe, inexpensive and scalable alternative to inducing antigen-specific immune responses [1]. DNA vaccines are a relatively modern approach, in which a plasmid encoding an antigen is expressed by the host in order to generate a protective immune response. DNA vaccines are considered safer than live-attenuated or whole pathogen vaccines due to their inability to cause disease. Despite their excellent safety profile, DNA-based formulations often lack immunogenicity and suffer rapid nuclease degradation and phagocytic elimination by the reticulo-endothelial system [2].

Nanoparticle delivery systems are being increasingly applied to improve the efficacy of DNA vaccines. The linking of DNA to a particulate carrier has been demonstrated to improve DNA vaccine uptake, expression and immune priming [1]. Nanoparticle-based carrier systems also afford protection to vaccine antigens from proteases and endonucleases, resulting in higher levels of antigen being taken up by immune cells in the body [3].

Particulate carrier systems have shown great potential as mediators for the delivery of vaccine antigens in vivo [4]. Nanoparticle-based nucleic acid delivery platforms based on metals such as gold and silver, and metal oxides such as silica and iron oxide, have been demonstrated to offer advantages over traditional soluble antigen vaccines [5,6]. Nanoparticle vaccine formulations can protect antigens from degradation resulting in greater immunological priming and improved immunological outcomes, as well as having adjuvant-like stimulatory properties themselves [2,3]. Some disadvantages of particulate carrier systems include poor clearance and the induction of non-specific inflammation which can interfere with adaptive immune responses [7]. Biocompatible nanoparticles such as those made from protein, organic polymer or lipid offer a safer alternative for antigen delivery [8,9].

Lipid nanoparticles are biocompatible nanocarriers which have been widely developed for the delivery of therapeutics and vaccine antigens due to their excellent safety profile and capacity to carry both lipophilic and hydrophilic payloads [10]. Lipid nanoparticles offer many benefits as vaccine delivery vehicles; they are regarded as safer than viral vectors given that they lack viral proteins, and are well tolerated in vivo due to their biocompatibility [11].

Cationic solid lipid nanoparticles have been widely applied for gene delivery by condensing and delivering negatively charged DNA or RNA to form nanostructured complexes called ‘lipoplexes’. Solid lipid nanoparticle lipoplexes have been demonstrated to effectively deliver siRNA and plasmid DNA in vitro [9,12,13,14,15,16] but there are no reports of adjuvant-modified solid lipid nanoparticles (SLNs) as a DNA vaccine delivery system. Monophosphoryl lipid A (MPL-A) is a non-toxic chemically modified lipopolysaccharide (LPS) analogue which acts as a potent Toll-like receptor 4 (TLR4) agonist, and has been licensed for use in commercial vaccines Ceravix (Human Papillomavirus) and Shingrix (Herpes zoster virus) [17,18]. The incorporation of MPL-A as an adjuvant lipid to the SLN-A nanoparticles affords an immunogenic structural component of the lipoplex complex, which may increase antigen-specific immune responses in vivo.

The aim of the experiments reported here is to synthesise and characterise adjuvanted SLN-A nanoparticles as a novel DNA vaccine delivery system by determining the stability, DNA loading capacity, and cellular uptake of nanoparticles, and by measuring TLR4 stimulation by expression of tumor necrosis factor alpha (TNF-α), the expression of encoded antigen, and cytotoxic effects of the nanocarrier platform on human embryonic kidney (HEK)293T cells. This study demonstrates the application of SLN-A nanoparticles as a nanocarrier system for a DNA vaccine encoding the *H. pylori* antigen urease alpha.

## 2. Materials and Methods

### 2.1. Synthesis of SLN-A Nanoparticles

SLN-A nanoparticles were prepared using a modified solvent-emulsification method [16]. Particles were synthesised as previously described [15] with a modification to include the adjuvant lipid monophosphoryl lipid A from Salmonella enterica serotype Minnesota Re 595 (Sigma-Aldrich, St. Louis, MO, USA). Briefly, cholesteryl oleate (45% *w*/*w*; Sigma-Aldrich, St. Louis, MO, USA), glyceryl trioleate (3% *w*/*w*; Sigma-Aldrich, St. Louis, MO, USA), DOPE (1,2-Dioleoyl-sn-glycero-3-phosphoethanolamine, 3-sn-phosphatidylethanolamine; 14% *w*/*w*; Sigma-Aldrich), cholesterol (10% *w*/*w*; Sigma-Aldrich, St. Louis, MO, USA), DC-cholesterol (3ß-[N-(N’,N’-dimethylaminoethane)-carbamoyl]cholesterol hydrochloride; 28% *w*/*w*; Sapphire Biosciences, Redfern, NSW, Australia) and lipid A, monophosphoryl, (0.5% *w*/*w*; Sigma-Aldrich, St. Louis, MO, USA) were dissolved in a chloroform/methanol mixture (2:1, *v*/*v*). Once dissolved, 10 mL of Milli-Q^®^ system-filtered ultrapure water (MQH2O; Merck, Fort Kenilworth, NJ, USA) was added and the suspension was vortexed thoroughly for 5 min. A final concentration of 5 mg/mL of lipid stock suspension was sonicated using a Branson digital sonicator, for 3 min, 40% duty cycle and 35% power output. The solvent was then removed from the microemulsion at 55 °C using a vacuum concentrator. Following synthesis, particle size and zeta potential were measured by dynamic light scattering (DLS), and particles were stored at 4 °C for up to 1 week before being used in the preparation of lipoplex-A.

### 2.2. Synthesis of Mammalian Plasmid Vaccine pcDNA3.1-UreA

A mammalian expression vector pcDNA3.1(-) encoding 717 bp *H. pylori* antigen Urease alpha (GenBank: AF373569.1) [19] was synthesised by Genscript (Piscataway, NJ, USA). The 5782 bp construct was amplified in *Escherichia coli* strain DH5α and purified using the QIAprep Spin Miniprep Kit (Qiagen, Germantown, MD, USA) and stored at −20 °C until use.

### 2.3. Synthesis of Lipoplex-A

Particles were resuspended in sterile phosphate buffered saline (PBS) by mixing vigorously for 1 min before the addition of plasmid DNA (pcDNA3.1-UreA, 5.8 kbp) synthesised as a DNA vaccine candidate encoding the *H. pylori* antigen urease alpha (UreA). The SLN and plasmid suspension was incubated at 4 °C overnight, after which the synthesised lipoplex-A samples were utilised for experimental analysis.

### 2.4. Characterisation of SLN-A and Lipoplex-A by TEM

The SLN-A nanoparticles and lipoplex-A complexes were characterised for size and morphology by transmission electron microscopy (TEM). Ten micrograms of SLN-A or lipoplex-A were loaded onto 200-mesh formvar–carbon copper grids and allowed to dry before visualising at 80 kV on a JEOL 1010 TEM (Akishima, TYO, Japan) using the Gatan Microscopy Suite software version 2.3 (Pleasanton, CA, USA).

### 2.5. Characterisation of SLN-A Stability

To assess the stability, SLN-A nanoparticles were stored at −20 °C, 4 °C or 22 °C (ambient temperature) in PBS for up to 4 weeks. At each timepoint the hydrodynamic size and surface zeta potential of the particles were measured by DLS (Zetasizer Nano ZS, Malvern Panalytical, Malvern, WR, UK).

### 2.6. DNA Binding and DNase Protection Assay

Agarose gel electrophoresis was used to study DNA binding and DNase protection afforded by lipoplex-A to plasmid DNA. Increasing weight ratios of lipoplex-A (1:1, 1:5, 1:10, 1:50, and 1:100 of DNA:SLN-A w/w) were incubated overnight at 4 °C. After binding, 10 µL of each sample was run per lane on a 1% agarose gel at 100 kV for 60 min. To determine the ability of the SLN-A to protect bound plasmid DNA from nuclease degradation, a DNase protection assay was performed using the same samples, which were treated with 1 U/µg of DNase I (New England Biolabs, Ipswich, MA, USA) for 30 min at 37 °C followed by heat inactivation of DNase I at 75 °C for 10 min. Following this, the remaining DNA was released from the lipoplexes by the addition of 10 µL of 1% SDS and samples were run on a 1% agarose gel to visualise the quantity of undigested plasmid DNA. Treated pcDNA3.1-UreA served as an internal positive control for DNase digestion.

### 2.7. Cell lines and Culture Conditions

The murine dendritic cell line DC2.4 was provided by Dr. Dodie Pouiniotis, Royal Melbourne Institute of Technology (RMIT) University. DC2.4 cells were cultured in Roswell Park Memorial Institute (RPMI) 1640 GlutaMAX™ media supplemented with 25 mM HEPES (4-(2-hydroxyethyl)-1-piperazineethanesulfonic acid) buffer and 100 IU/mL penicillin-100 µg/mL streptomycin and 10% filter-sterilised heat-inactivated fetal bovine serum (FBS) (all sourced from Gibco, Thermo Fisher Scientific, Scoresby, VIC, Australia). HEK293T human embryonic kidney cells were provided by Dr. Brett Cromer, Swinburne University of Technology. HEK293T cells were cultured in Dulbecco’s Modified Eagle Medium (DMEM) media supplemented with 100 IU/mL penicillin-100 µg/mL streptomycin and 10% filter-sterilised heat-inactivated FBS (Complete DMEM). The human monocyte cell line Tohoku Hospital Pediatrics-1 (THP-1) was provided by Dr. Anna Walduck, RMIT University. THP-1 cells were cultured in DMEM media supplemented with 100 IU/mL penicillin−100 µg/mL streptomycin and 10% filter-sterilised heat-inactivated FBS (Complete DMEM). All cell lines were maintained at 37 °C in a 5% CO_2_ humidified atmosphere and passaged at 70–90% confluency.

### 2.8. Evaluation of TNF-α Expression in THP-1 cells Stimulated with SLN-A

To determine the ability of SLN-A particles to act as a TLR4 agonist, the expression of proinflammatory cytokine tumor necrosis factor alpha (TNF-α) by human macrophage-like THP-1 cells was measured. THP-1 cells were activated with 100 nM phorbol 12-myristate 13-acetate (PMA) (Sigma-Aldrich, St. Louis, MO, USA) for 24 h before exposure to 100 µg/mL SLN-A nanoparticles for 6 h. Treatment with media alone or heat inactivated *E. coli* DH5α lysate served as negative and positive controls, respectively. After 6 h exposure, cell supernatant was collected and the expression of TNF-α was measured using a LEGEND MAX™ Human TNF-α ELISA Kit (Biolegend, San Diego, CA, USA) as per the manufacturer’s instructions.

### 2.9. Evaluation of the Cytotoxicity of SLN-A and Lipoplex-A

The cytotoxicity of SLN-A and lipoplex-A was evaluated at a range of concentrations, using PrestoBlue^®^ Cell Viability reagent (Invitrogen, Thermo Fisher Scientific, Scoresby, VIC, Australia) according to the manufacturer’s instructions. Briefly, HEK293T cells were seeded in a 96 well plate at a density of 5 × 10^3^ cells per well and incubated at 37 °C in 5% CO_2_ humidified atmosphere overnight. Subsequently, media were replaced by 100 μL fresh complete DMEM containing a final concentration of 5, 10, 25, 50, 75 or 100 μg/mL SLN-A or lipoplex-A and incubated for 24, 48 or 72 h. Untreated cells served as control for baseline cell viability. Cell viability was normalised by using the untreated control as the 100% cell viability.

### 2.10. Characterisation of SLN-A Uptake and Sub-Cellular Localisation by TEM

Uptake and intracellular localisation of SLN-A by DC2.4 cells was visualised by TEM. The murine dendritic cell line DC2.4 cells was used for this study in order to characterise the uptake pathway by key antigen presenting cells which are known to initiate the adaptive immune response. Ninety nanometre-thick cell sections of fixed and resin embedded DC2.4 cells with internalised SLN-A nanoparticles were viewed by TEM, as previously described [20]. Briefly, cells were treated with SLN-A for 6 h before being fixed overnight in a primary fixative of 2.5% glutaraldehyde, 2% paraformaldehyde (PFA) in 100 mM cacodylate buffer (pH 7.4), followed by three washes with 100 mM cacodylate buffer. Subsequently, cells were post-fixed with a secondary fixative of 1% osmium tetroxide and 1.5% potassium ferricyanide for 1.5 h, followed by three washes with distilled water. Samples were then dehydrated with increasing gradients of ethanol (50%, 70%, 90%, 95% and 100%) for 15–30 min. Following this, cells were vacuum-infiltrated and embedded in Spurr’s resin [20] before ultramicrotome sectioning to a thickness of 90 nm using a Leica Ultracut UCT ultramicrotome (Wetzlar, Hesse, Germany). The sections were loaded onto holey-carbon grids and observed at 80 kV by a JEOL 1010 TEM using the Gatan Microscopy Suite software version 2.3.

### 2.11. Characterisation of SLN-A Uptake by Fluorescence Microscopy

To observe cellular uptake of SLN-A, particles were fluorescently tagged by the addition of fluorescently labelled phospholipid (1,2-dioleoyl-sn-glycero-3-phosphoethanolamine-N-(lissamine rhodamine B sulfonyl)) (0.5% w/w; Avanti Polar Lipids, Alabaster, AL, USA) prior to sonication during SLN-A synthesis. DC2.4 cells were treated with fluorescently tagged SLN-A nanoparticles for up to 24 h and imaged by fluorescence microscopy. Cells were seeded onto 25 mm glass coverslips in a 6 well plate overnight at a density of 2 × 10^5^ cells per well. Cells were treated with 100 μg SLN-A in complete RPMI 1640 GlutaMAX™ medium at 37 °C for 0, 1, 3, 6, 12 or 24 h to allow cellular uptake of the nanoparticles. Untreated cells served as a negative control. Following incubation, unbound nanoparticles were removed by washing with cold PBS three times. Cells were then permeabilized by adding 0.1% Triton X-100 with 4% PFA for 15 min at room temperature, followed by three washes with cold PBS. Nuclear staining with DAPI (4′,6-diamidino-2-phenylindole; Invitrogen, Thermo Fisher Scientific, Scoresby, VIC, Australia) was performed at room temperature for 15 min, followed by three washes with cold PBS. The coverslips were carefully mounted and sealed onto a glass slide using ProLong™ Gold Antifade Mountant (Invitrogen, Thermo Fisher Scientific, Scoresby, VIC, Australia) and imaged using a Leica Epifluorescence microscope.

### 2.12. Confirmation of Lipoplex-A Transfection

To confirm the ability of lipoplex-A to transfect murine immune cells in vitro, DC2.4 cells were transfected with lipoplex-A for 72 h, and recombinant UreA protein expression was visualised by fluorescence microscopy. Briefly, DC2.4 cells were seeded onto 25 mm glass coverslips in a 6 well plate at a density of 2 × 10^5^ cells per well and incubated at 37 °C in a 5% CO_2_ humidified atmosphere for 24 h. Following this, 100 μg of lipoplex-A was added to each well and incubated at 37 °C for up to 72 h to allow cellular uptake of the lipoplexes and expression of recombinant UreA. Following incubation, the unbound SLN were removed by gentle washing with PBS three times. Cells were then fixed and permeabilized by adding 0.1% Triton X-100 with 4% PFA for 15 min. Subsequently, nuclear staining with DAPI was performed at room temperature for 15 min, followed by three washes with PBS. The cells were then probed for UreA protein expression with AlexaFluor 488 conjugated anti-6XHis monoclonal antibody (Abcam, Cambridge, CB, UK) suspended in 1% bovine serum albumin (BSA) (1:100) at room temperature for 60 min, followed by three washes with PBS. The coverslips were carefully mounted and sealed onto a glass slide using gold antifade reagent. Cellular imaging was performed using a Leica Epifluorescence microscope (Wetzlar, Hesse, Germany).

### 2.13. Statistical Analysis

All the experiments were carried out in triplicate and the results were expressed as the mean ± standard deviation. Analysis of results was performed by two-way ANOVA with multiple comparisons using GraphPad Prism software (version 8; GraphPad Software, San Diego, CA, USA).

## 3. Results

### 3.1. Characterisation of SLN-A

Solid lipid nanoparticles were successfully synthesised by a modified solvent-emulsification method [16] adapted to include the addition of adjuvant lipid MPL-A, producing novel adjuvanted SLN-A nanoparticles. Freshly synthesised SLN-A were found to have an average zeta potential of 57.5 ± 6.9 mV.

### 3.2. Characterisation of SLN-A and Lipoplex-A Size and Morphology

**Figure 1:**
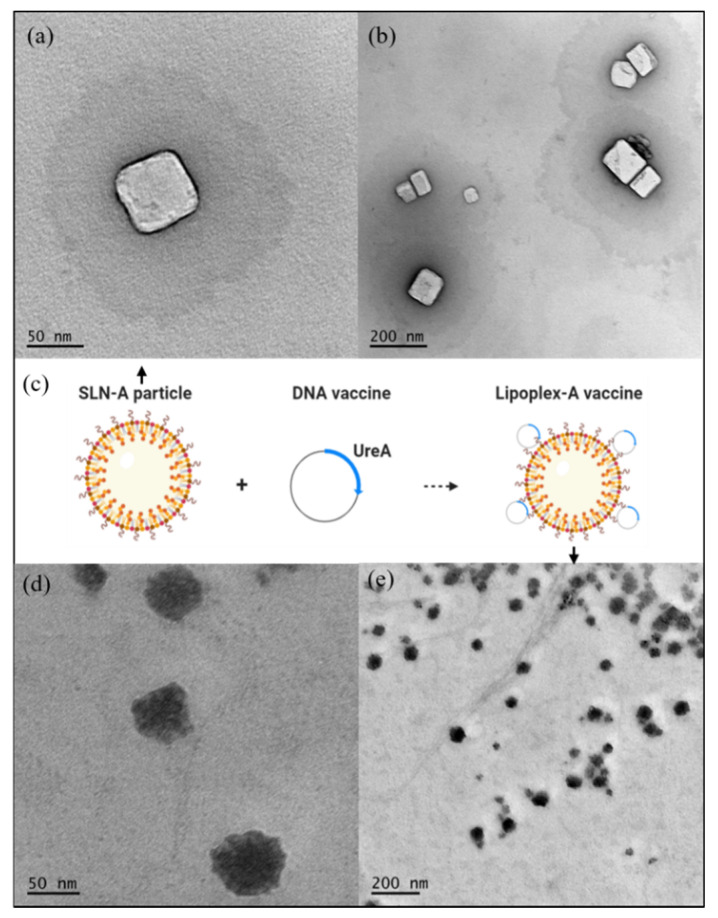
TEM micrographs under 100k accelerating voltage of adjuvanted solid lipid nanoparticle (SLN-A) nanoparticles at (**a**) 50 k magnification and (**b**) 20 k magnification; (**c**) a schematic representation of SLN-A particles binding DNA to form lipoplex-A; (**d**) lipoplex-A imaged at 50 k magnification and (**e**) 20 k magnification. SLN-A nanoparticles were found to be heterogenous in size and cuboid in shape with an average diameter of 75.63 ± 31.99 nm, while lipoplex-A complexes were heterogenous in size with undefined edges and had an average diameter of 74.67 ± 39.93 nm.

TEM micrographs showed SLN-A nanoparticles to have an average diameter of 75.63 ± 31.99 nm (Figure 1a,b). Particles appeared heterogenous in size, with most of the particle population being cuboid in shape. Particles were loaded with plasmid DNA to form lipoplex-A (Figure 1c) which were found to be heterogenous in size and shape with undefined edges and an average diameter of 74.67 ± 39.93 nm (Figure 1d,e).

### 3.3. Characterisation of SLN-A Stability

**Figure 2:**
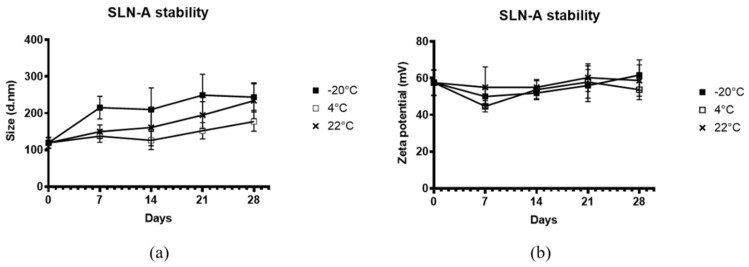
Characterisation by dynamic light scattering (DLS) of stability of SLN-A (**a**) particle size; (**b**) zeta potential at −20 °C, 4 °C or 22 °C for up to 4 weeks. The effect of temperature on particle size and zeta potential was determined not to be statistically significant by two-way ANOVA (F(2,6) = 4.686, *p* = 0.0595). However, the effect of storage time was determined to be statistically significant for both particle size (F(2.163, 12.98) = 19.02, *p* = 0.0001) and zeta potential (F(1.679, 10.07) = 4.780, *p* = 0.0393).

To determine the stability of the adjuvanted SLN-A, particles were stored at −20 °C, 4 °C or 22 °C for up to 4 weeks. At each timepoint particle size and zeta potential were measured by DLS (Figure 2). Particles stored at −20 °C were found to have a greater hydrodynamic size and were more heterogenous in size at all timepoints after the initial measurement, while particles stored at 4 °C and 22 °C appeared to remain stable in size over the 4-week period (Figure 2a). Fluctuations in the zeta potential of particles were observed across all three temperature conditions over the four-week period (Figure 2b). The effect of temperature on particle size and zeta potential was not found to be statistically significant by two-way ANOVA (F(2,6) = 4.686, *p* = 0.0595). However, the effect of storage time was determined to be statistically significant for both particle size (F(2.163, 12.98) = 19.02, *p* = 0.0001) and zeta potential (F(1.679, 10.07) = 4.780, *p* = 0.0393), suggesting that particle stability is time-dependent, but not temperature-dependent.

### 3.4. DNA Binding and DNase Protection

**Figure 3:**
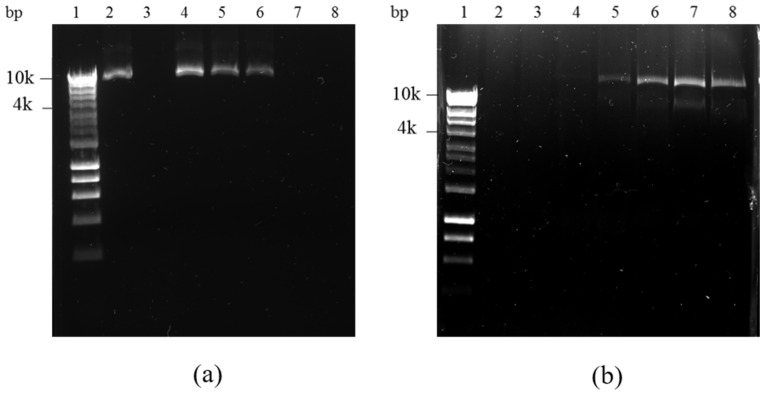
Agarose gel electrophoresis of a range of DNA:SLN-A weight ratios. (**a**) DNA binding assay: Lane 1—Hyperladder I, lane 2—plasmid DNA only, lane 3: SLN-A only, lane 4–8: 1:1, 1:5, 1:10, 1:50, 1:100 DNA:SLN weight ratio lipoplexes; (**b**) DNase I protection assay: Lane 1—Hyperladder I, lane 2—digested plasmid DNA, lane 3: SLN-A only, lane 4–8: 1:1, 1:5, 1:10, 1:50, 1:100 DNA:SLN weight ratio lipoplexes treated with DNase I and subsequently released from particles by SDS treatment.

Lipoplex-A formulations composed of increasing DNA:SLN-A weight ratios were characterised by electrophoresis in an agarose gel to determine the binding capacity of the SLN-A, showing inhibition of DNA movement through the gel due to high intensity binding by the nanoparticles (Figure 3). To determine the ability of SLN-A nanoparticles to protect plasmid DNA from DNase enzyme degradation, all samples were treated with DNase I and DNA released from SLN-A for visualisation of protected DNA. Unbound DNA from SLN-A based lipoplexes synthesised at RT was electrophoresed through the gel (Figure 3a) and binding of DNA by lipoplex-A is demonstrated by a reduction of DNA band intensity compared to unbound plasmid DNA (Figure 3a, lane 2). Complete plasmid loading by SLN-A particles was evident at a 1:50 DNA:SLN-A weight ratio (Figure 3a, lane 7), suggesting an approximate DNA loading capacity of 20 µg DNA/mg of SLN-A. No visual interference was caused by SLN-A in the gel (Figure 3a, lane 3). Plasmid DNA was released from SLN-A particles after lipoplexes were subject to DNase digestion and released by SDS (Figure 3b). Higher concentration of bound DNA was indicated by stronger band intensity, which was protected from DNase degradation. Significant plasmid protection was observed at ratios of 1:50 and 1:100 DNA:SLN-A (Figure 3b, lane 7–8). Undigested plasmid DNA were also evident at lower weight ratios (Figure 3b, lanes 5–6), indicating that SLN-A binds and protects plasmid DNA at ratios as low as 1:5, while no protection was evident at 1:1 ratio (Figure 3b, lane 4).

### 3.5. Evaluation of TNF-α Expression in THP-1 Cells Stimulated with SLN-A

**Figure 4:**
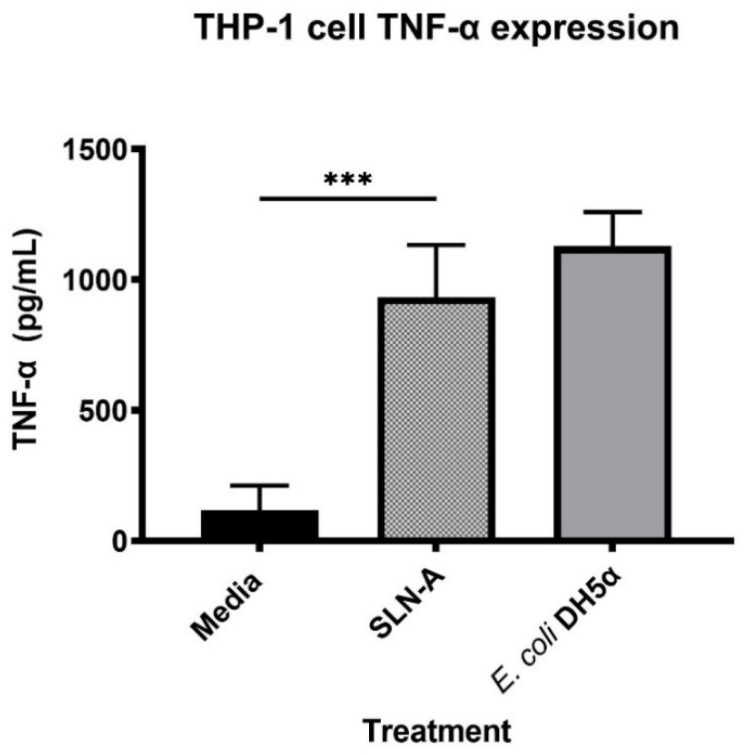
Expression of tumor necrosis factor alpha (TNF-α) by THP-1 cells stimulated with SLN-A particles. One-way ANOVA confirmed that a significant difference in TNF-α expression was observed between cells treated with media and cells treated with SLN-A (F(2,6) = 39.35, *p* = 0.0009). *** denotes *p* ≤ 0.001.

The ability of SLN-A particles to act as a TLR4 agonist by stimulating expression of pro-inflammatory cytokine TNF-α was measured (Figure 4). THP-1 cells were found to secrete significantly higher levels of TNF-α in response to SLN-A than to media alone (F(2,6) = 39.35, *p* = 0.0009). No significant difference in TNF-α expression was detected between cells treated with SLN-A compared to cells treated with *E. coli* DH5α lysate which served as the positive control.

### 3.6. Evaluation of the Cytotoxicity of SLN-A and Lipoplex-A

**Figure 5:**
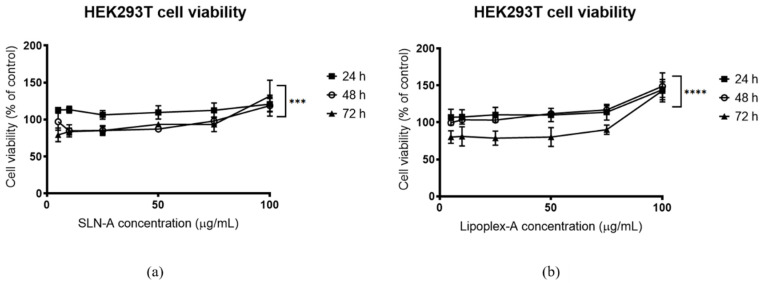
Viability of human embryonic kidney (HEK)293T cells treated with (**a**) SLN-A nanoparticles; (**b**) lipoplex-A (SLN-A with loaded plasmid DNA) for 24, 48 or 72 h. Cell viability increased across both treatments in a dose dependent manner across all three timepoints. Cell viability was lowest for cells treated with lipoplex-A for 72 h. Two-way ANOVA confirmed that a significant difference in cell viability was observed between timepoints for cells treated with both SLN-A (F(1.279, 15.35) = 21.24, *p* = 0.0002) and lipoplex-A (F(1.222, 14.66) = 32.56, *p* = < 0.0001). *** denotes *p* ≤ 0.001, and **** denotes *p* ≤ 0.0001.

The cytotoxicity of SLN-A and lipoplex-A against HEK293T cells was determined for up to 72 h with either SLN-A or lipoplex-A. Cell viability was maintained above 70% at all concentrations of SLN-A (Figure 5a) and Lipoplex-A (Figure 5b) for all time points. At all timepoints for both SLN-A and lipoplex-A treatments, the highest concentration treatment (100 µg/mL) resulted in high cell viability. Two-way ANOVA was used to confirm that a significant difference in cell viability was observed between timepoints for cells treated with both SLN-A (F(1.279, 15.35) = 21.24, *p* = 0.0002) and lipoplex-A (F(1.222, 14.66) = 32.56, *p* = <0.0001). Cells treated for 72 h had the lowest viability, however viability was maintained above 70% at all treatment concentrations, indicating negligible cytotoxicity by SLN-A and lipoplex-A formulations.

### 3.7. Characterisation of SLN-A Uptake and Localisation by TEM

**Figure 6:**
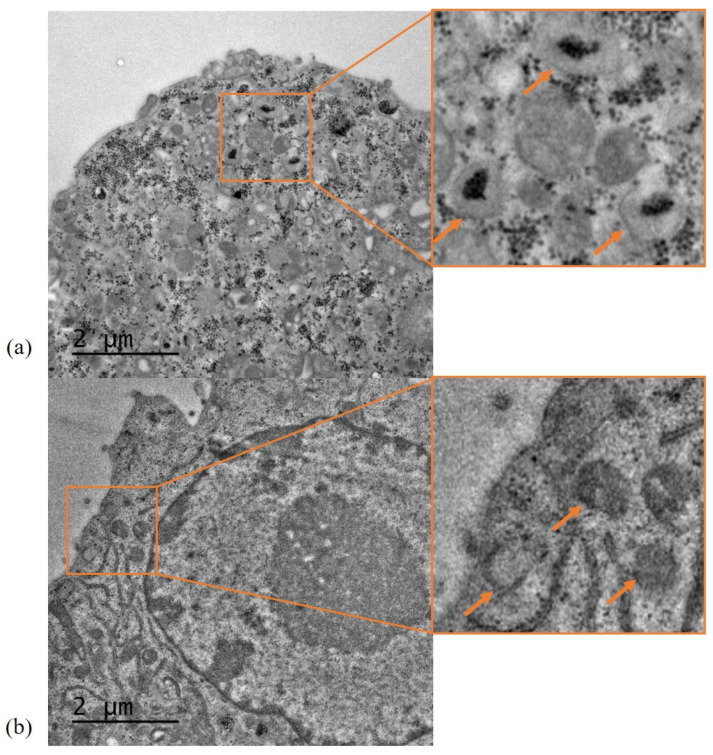
TEM micrographs of SLN-A nanoparticle uptake by DC2.4 cells. (**a**) Cells treated with SLN-A nanoparticles; (**b**) Control cells without nanoparticle treatment. Nanoparticle uptake was evident within cells treated with SLN-A nanoparticles (image a), with aggregation in endosomes (inset). No nanoparticle uptake was seen in the control cell sample (image b), where empty endosomes were observed (inset). Scale bar 2 µm.

Internalisation of SLN-A particles was characterised using DC2.4 murine dendritic cells. Cells treated with SLN-A were found to contain SLN-A encapsulated within endosomal compartments (indicated by orange arrows) (Figure 6a). Control cells appear different to treated cells, being visibly free of nanoparticles (Figure 6b).

### 3.8. Characterisation of SLN-A Uptake by Fluorescence Microscopy

**Figure 7:**
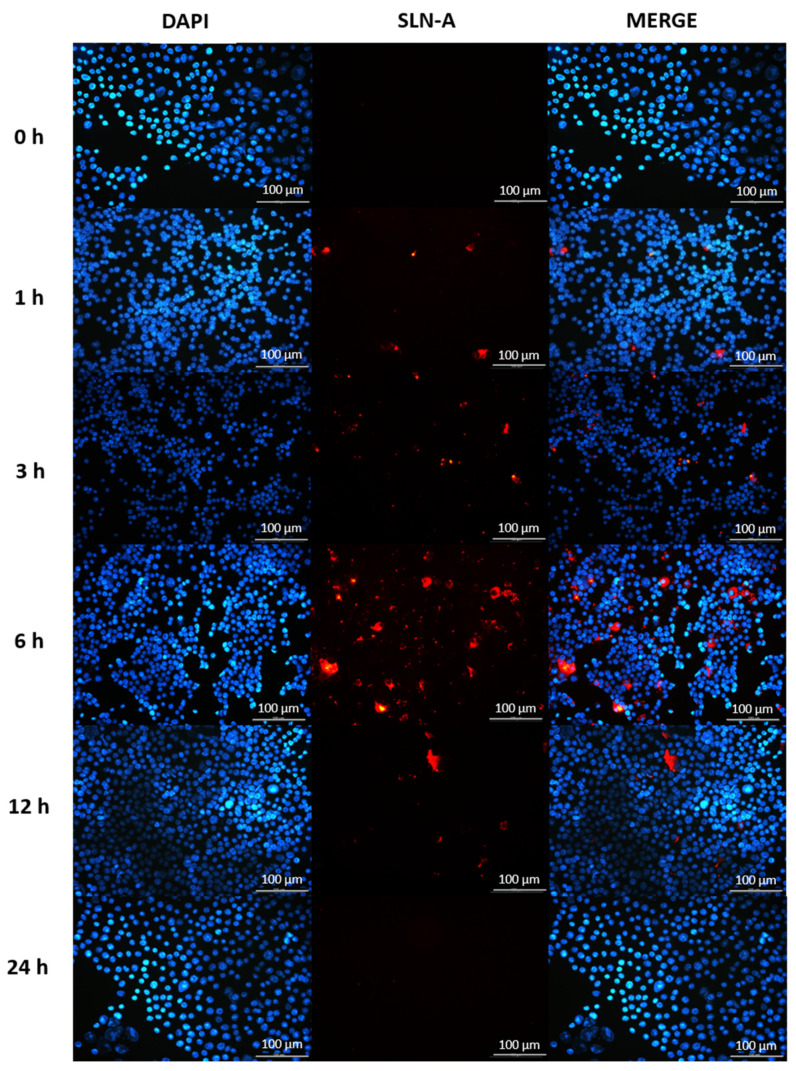
Fluorescence micrographs of DC2.4 cells internalising SLN-A nanoparticles within 24 h. Cell nuclei counterstained with DAPI (blue) and SLN-A nanoparticles (red) are displayed. The speed of cell uptake of SLN-A nanoparticles by DC2.4 cells was visualised at 0, 1, 3, 6, 12 and 24 h timepoints. Particles were internalised from 3 h and were no longer visible within the cell by 24 h. Scale bar 100 µm.

The speed of cellular uptake of SLN-A nanoparticles was determined in DC2.4 cells exposed to SLN-A for up to 24 h by fluorescence microscopy. DAPI-stained cell nuclei are shown in blue, and particles are visible in red. DC2.4 were found to internalise SLN-A within 3 h, with maximum particle uptake at 6 h and an absence of internalised particles at 24 h (Figure 7).

### 3.9. Confirmation of Lipoplex Transfection of Murine Dendritic Cells

**Figure 8:**
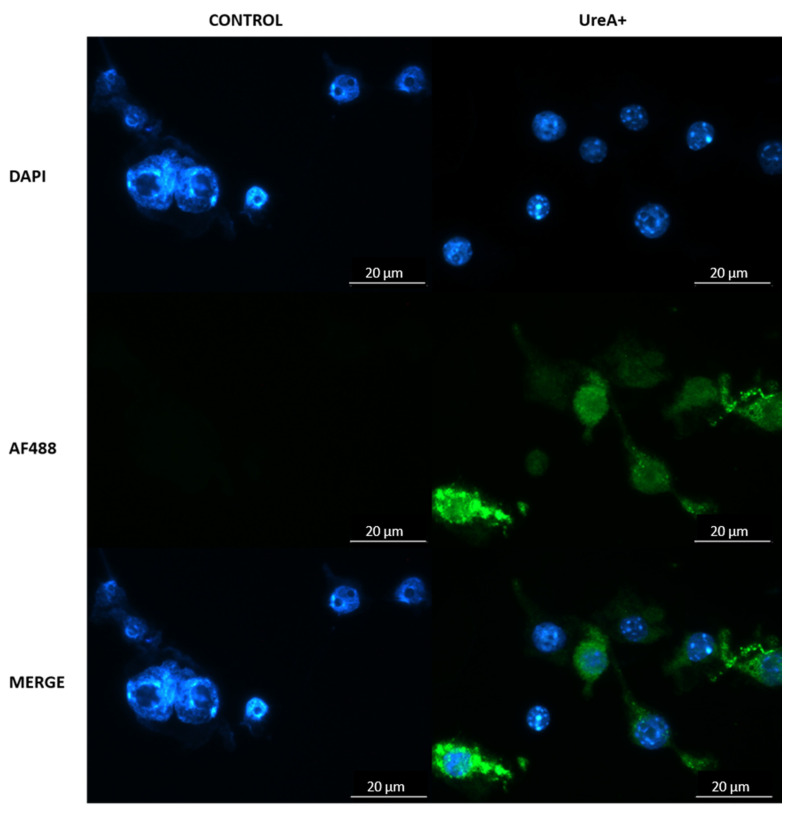
Fluorescence micrograph of DAPI-stained DC2.4 cells (blue; nuclei) expressing Urease alpha (UreA) labelled with AlexaFluor 488 conjugated anti-His monoclonal antibody (green). Cells were transfected with lipoplex-A (SLN-A nanoparticles loaded with pcDNA3.1-UreA) and were found to be expressing UreA protein indicated by the binding of anti-6XHis antibody. Scale bar 20 µm.

Efficient transfection of murine immune cells by lipoplex-A and recombinant UreA protein expression were confirmed by fluorescence microscopy at 72 h. DC2.4 cells transfected with lipoplex-A carrying pcDNA3.1-UreA readily expressed recombinant UreA protein (green) in vitro detected by fluorescently tagged anti-His monoclonal antibody (green; Figure 8).

## 4. Discussion

Low transfection rates due to endonuclease degradation and poor cellular uptake are major barriers to the clinical application of DNA vaccines. However, the delivery of DNA vaccines by a nanoparticle carrier can improve stability and offer protection to vaccine antigens in vivo, allowing increased cell uptake and expression of encoded antigens [21]. In this study, novel cationic SLN-A nanoparticles containing the adjuvant lipid MPL-A were synthesised to serve as a delivery system for a DNA vaccine against *H. pylori*. MPL-A is an attenuated analogue of lipopolysaccharide (LPS) and a potent TLR4 agonist, making it an ideal vaccine antigen and suitable lipid species for incorporation into a SLN formulation [22]. Cationic SLN are a prominent class of nanomaterials with potential applications in delivery of nucleic acids due to their high transfection capacity and surface binding of anionic nucleic acids [23,24].

Synthesised SLN-A nanoparticles were heterogenous in size and cuboid in shape, with a cationic surface charge. Particles previously synthesised by the emulsification method have been reported to be polydisperse and hexagonal shaped, with a similar size and surface charge [15]. Lipid nanoparticles are highly diverse in size, owing to their affinity for electrostatic interaction and aggregation. There are numerous variables which can cause high variability in SLN polydispersity, particularly the concentration of key lipid species, storage temperature and synthesis method [25]. Lipoplex-A particle-DNA complexes were found to be of similar size but were notably more heterogenous in shape, with undefined edges. Previous studies have found that nanoparticle shape and size change upon DNA loading, which may be due to complete coating of nanoparticles thus obscuring the edges and giving the lipoplexes a quasi-spherical appearance [14,26]. Lipoplex-A samples also contained small aggregates of DNA visible as smaller particle contaminants in the sample, assumed to be a product of the TEM preparation process, owing to the wetting effect of the hydrophobic grid surface on unbound hydrophilic DNA as previously observed [27].

Previously, SLN developed by Kim et al. were synthesised with a cationic lipid species, DC-Cholesterol, which provides a positive charge to the particle surface, enabling them to bind DNA electrostatically [16]. SLN nanoparticles produced by this method have been applied to delivery of DNA and siRNA as therapeutics but have not previously been modified to include an adjuvant to serve as a DNA vaccine nanocarrier platform [15,16]. SLN-A nanoparticles from the present study bound DNA completely at a weight ratio of 1:50 (w/w) DNA:SLN-A, indicating a loading capacity of 20 µg DNA/mg SLN-A. SLN-A particles also demonstrated their ability to bind and protect DNA from DNase degradation. The ability of lipid nanoparticles to protect DNA from nuclease degradation has been documented and is attributed to strong electrostatic interaction with DNA at the particle surface, preventing access to and degradation of bound DNA by nucleases [12,14,15].

Stability of SLN-A nanoparticles was independent of temperature, with no differences observed between groups at all three temperatures. Particle stability was however significantly affected by storage time, with differences in particle size and zeta potential observed across the four-week storage period, particle stability therefore appears to be time-dependent but not temperature-dependent. Previous SLN stability studies have demonstrated the stability of SLN nanoparticles at 4 °C with reduced stability at room temperature (RT) [28]. Typically, lipids are more thermostable at low temperatures, but individual lipid species have unique phase transition temperatures (T_m_) governed by a number of factors including hydrocarbon length, saturation and charge. Despite the high T_m_ of core lipid component cholesterol oleate (47 °C), the stability of SLN-A may be affected by the low T_m_ of helper lipid DOPE (−16 °C) which forms a significant proportion (14% w/w) of the original lipid emulsion used to synthesise SLN-A nanoparticles.

The main lipid species of these SLN-A nanoparticles is cholesterol-oleate which has a melting temperature of 47 °C, yielding SLN-A nanoparticles which are solid at ambient and body temperatures. Due to their solid core, SLN have higher stability than liposomes and microemulsions and therefore offer some benefits in biological applications, such as targeted release of drugs or antigen, and stability at body temperature [8]. Cholesterol is a major lipid component of cell membranes, facilitating mechanical strength and elasticity of membranes, and stabilising membrane/nanoparticle interaction. DC-Cholesterol is the cationic lipid species used to afford a positive charge to the particles, promoting binding and condensation of negatively charged DNA.

Previous studies have determined that MPL-A induces secretion of pro-inflammatory cytokine TNF-α through TLR4-dependant signalling events [29]. The ability of SLN-A to stimulate the production of TNF-α at a level equivalent to bacterial derived LPS demonstrates the ability of the particles to act as TLR4 agonists and highlights the adjuvant properties of the particle platform.

Solid lipid nanoparticles are regarded as highly biocompatible formulations suitable for sensitive biological applications such as ocular and intranasal drug delivery [8]. SLN-A nanoparticles did not appear to exert toxicity towards HEK293T cells over 72 h of exposure. At high SLN-A and lipoplex-A concentrations an increase in cell proliferation was detected, likely driven by high metabolic activity due to the ready availability of cholesterol in the particle formulation. Studies have demonstrated that cholesterol promotes cellular uptake of Ca^2+^ by activation of the protein kinase B (AKT) pathway, and promotes cell growth and proliferation in vitro [30], and therefore it is likely that high cell viability is driven by increased Ca^2+^ uptake and availability of cholesterol as a key membrane component.

Cellular uptake of the SLN-A nanoparticles occurred within 3 h, and with maximum internalisation by 6 h. Particles were not visible within the cell by 24 h, indicating endosomal processing and metabolism of particles. Co-localisation of SLN-A within endosomes was evident within cell sections in Figure 6, which was previously observed for SLN nanoparticles lacking MPL-A [15]. Transfection of immune cells by lipoplex-A in vitro was evident after 72 h, demonstrating effective delivery of bound plasmid DNA to the nucleus for the transfection and expression of recombinant UreA protein. Cationic lipid particles improve the ability of nucleic acids to complete endosomal escape by neutralising the charge differential between DNA and endosomal membrane [31]. Endosomal escape is a necessary step for the transfection of cells and expression of DNA vaccine antigens in vivo, thus SLN-A nanoparticles may serve as an effective delivery vehicle for DNA vaccine antigens due to their strong cationic surface charge. Although commercially available liposomal transfection reagents such as Lipofectamine induce high levels of transfection in vitro, liposomal formulations suffer reduced stability and low encapsulation efficiency in vivo when compared to solid lipid nanoparticle formulations [32].

Both MPL-A and CpG (deoxycytidylate-phosphate-deoxyguanylate) motifs present in plasmid DNA have adjuvant activity and are capable of stimulating DC to produce pro-inflammatory cytokines and activate T cells [17,33]. Vaccine formulations with MPL-A+CpG have previously been demonstrated to be immunogenic [22]. The SLN-A nanoparticle platform offers a vaccine platform for DNA vaccines which can co-deliver MPL-A and CpG adjuvants through the binding of CpG containing DNA vectors. Surface modification has been shown to augment antigen-specific DC responses to nanovaccine carriers [34]. The design of particle-based vaccine nanocarriers must consider the influence of surface modification and vaccine cargo on resulting immune responses.

Lipid nanoparticle formulations are being developed with increasing tolerability and biocompatibility for the application of DNA and RNA-based vaccines [13]. Such formulations may improve the immunogenicity and efficacy of DNA vaccines in the future, by providing a safe and effective delivery vehicle for vaccine antigens. Lipid nanoparticles modified to include vaccine adjuvants have potential as biocompatible, surface-modifiable, and immunogenic delivery vehicles.

## 5. Conclusions

In conclusion, novel adjuvanted SLN-A nanoparticles were synthesised and characterised to determine size, zeta potential, stability, and cellular uptake. Particles were further characterised for TNF-α stimulation, DNA loading and DNase protection capacity. SLN-A nanoparticle lipoplexes were found to be non-cytotoxic and effective at transfecting mammalian immune cells in vitro and show promise for vaccine delivery.

## References

[1] Hobernik D., Bros M. (2018). DNA Vaccines-How Far From Clinical Use?. Int. J. Mol. Sci..

[2] Lim M., Badruddoza A.Z.M., Firdous J., Azad M., Mannan A., Al-Hilal T.A., Cho C.S., Islam M.A. (2020). Engineered Nanodelivery Systems to Improve DNA Vaccine Technologies. Pharmaceutics.

[3] Cai P., Zhang X., Wang M., Wu Y.L., Chen X. (2018). Combinatorial Nano-Bio Interfaces. ACS Nano.

[4] Taki A.C., Francis J.E., Skakic I., Dekiwadia C., McLean T.R., Bansal V., Smooker P.M. (2020). Protein-only nanocapsules induce cross-presentation in dendritic cells, demonstrating potential as an antigen delivery system. Nanomed. Nanotechnol. Biol. Med..

[5] Poddar A., Conesa J.J., Liang K., Dhakal S., Reineck P., Bryant G., Pereiro E., Ricco R., Amenitsch H., Doonan C. (2019). Gene Therapy: Encapsulation, Visualization and Expression of Genes with Biomimetically Mineralized Zeolitic Imidazolate Framework-8 (ZIF-8) (Small 36/2019). Small.

[6] Poddar A., Pyreddy S., Carraro F., Dhakal S., Rassell A., Field M.R., Reddy T.S., Falcaro P., Doherty C.M., Shukla R. (2020). ZIF Polymorphs for Nucleic Acid Delivery and Targeted Knockdown of Gene Expression in Prostate Cancer. ChemRxiv Prepr..

[7] Hua S., de Matos M.B.C., Metselaar J.M., Storm G. (2018). Current Trends and Challenges in the Clinical Translation of Nanoparticulate Nanomedicines: Pathways for Translational Development and Commercialization. Front. Pharm..

[8] Puri A., Loomis K., Smith B., Lee J.-H., Yavlovich A., Heldman E., Blumenthal R. (2009). Lipid-based nanoparticles as pharmaceutical drug carriers: From concepts to clinic. Crit. Rev. Ther. Drug Carr. Syst..

[9] Penumarthi A., Basak P., Smooker P., Shukla R., Daima H.K., Pn N., Ranjan S., Dasgupta N., Lichtfouse E. (2020). Hitching a Ride: Enhancing Nucleic Acid Delivery into Target Cells Through Nanoparticles. Nanoscience in Medicine.

[10] Rezaee M., Oskuee R.K., Nassirli H., Malaekeh-Nikouei B. (2016). Progress in the development of lipopolyplexes as efficient non-viral gene delivery systems. J. Control. Release.

[11] Zhao Y., Huang L. (2014). Lipid nanoparticles for gene delivery. Adv. Genet..

[12] Bondi M.L., Azzolina A., Craparo E.F., Lampiasi N., Capuano G., Giammona G., Cervello M. (2007). Novel cationic solid-lipid nanoparticles as non-viral vectors for gene delivery. J. Drug Target..

[13] Hassett K.J., Benenato K.E., Jacquinet E., Lee A., Woods A., Yuzhakov O., Himansu S., Deterling J., Geilich B.M., Ketova T. (2019). Optimization of Lipid Nanoparticles for Intramuscular Administration of mRNA Vaccines. Mol. Ther. Nucleic Acids.

[14] Karagöz U., Kotmakçı M., Akbaba H., Çetintaş V.B., Kantarcı G. (2018). Preparation and characterization of non-viral gene delivery systems with pEGFP-C1 Plasmid DNA. Braz. J. Pharm. Sci..

[15] Penumarthi A., Parashar D., Abraham A.N., Dekiwadia C., Macreadie I., Shukla R., Smooker P.M. (2017). Solid lipid nanoparticles mediate non-viral delivery of plasmid DNA to dendritic cells. J. Nanoparticle Res..

[16] Kim H.R., Kim I.K., Bae K.H., Lee S.H., Lee Y., Park T.G. (2008). Cationic solid lipid nanoparticles reconstituted from low density lipoprotein components for delivery of siRNA. Mol. Pharm..

[17] Wagner R., Hildt E. (2019). Composition and mode of action of adjuvants in licensed viral vaccines. Bundesgesundheitsblatt Gesundh. Gesundh..

[18] Del Giudice G., Rappuoli R., Didierlaurent A.M. (2018). Correlates of adjuvanticity: A review on adjuvants in licensed vaccines. Semin. Immunol..

[19] Chu C., Yu Y.J., Kong M.S., Ou J.T. (2003). Rate of Helicobacter pylori infection in children and clonality of Taiwan strains. Microbiol. Immunol..

[20] Spurr A.R. (1969). A Low-Viscosity Epoxy Resin Embedding Medium for Electron Microscopy. J. Ultrastruct. Res..

[21] Taki A., Kikidopolous N., Smooker P. (2011). Improving the Immunogenicity of DNA Vaccines.

[22] Ko E.J., Lee Y.T., Lee Y., Kim K.H., Kang S.M. (2017). Distinct Effects of Monophosphoryl Lipid A, Oligodeoxynucleotide CpG, and Combination Adjuvants on Modulating Innate and Adaptive Immune Responses to Influenza Vaccination. Immune Netw..

[23] Xiao Y., Shi K., Qu Y., Chu B., Qian Z. (2019). Engineering Nanoparticles for Targeted Delivery of Nucleic Acid Therapeutics in Tumor. Mol. Ther. Methods Clin. Dev..

[24] Parashar D., Rajendran V., Shukla R., Sistla R. (2020). Lipid-based nanocarriers for delivery of small interfering RNA for therapeutic use. Eur. J. Pharm. Sci..

[25] Bahari L.A.S., Hamishehkar H. (2016). The Impact of Variables on Particle Size of Solid Lipid Nanoparticles and Nanostructured Lipid Carriers; A Comparative Literature Review. Adv. Pharm. Bull..

[26] Yuan H., Zhang W., Du Y.Z., Hu F.Q. (2010). Ternary nanoparticles of anionic lipid nanoparticles/protamine/DNA for gene delivery. Int. J. Pharm..

[27] Gustafsson J., Arvidson G., Karlsson G., Almgren M. (1995). Complexes between cationic liposomes and DNA visualized by cryo-TEM. Biochim. Biophys. Acta.

[28] Wang Y., Zhu L., Dong Z., Xie S., Chen X., Lu M., Wang X., Li X., Zhou W. (2012). Preparation and stability study of norfloxacin-loaded solid lipid nanoparticle suspensions. Colloids Surf. B Biointerfaces.

[29] Okemoto K., Kawasaki K., Hanada K., Miura M., Nishijima M. (2006). A Potent Adjuvant Monophosphoryl Lipid A Triggers Various Immune Responses, but Not Secretion of IL-1β or Activation of Caspase-1. J. Immunol..

[30] Sun Y., Sukumaran P., Varma A., Derry S., Sahmoun A.E., Singh B.B. (2014). Cholesterol-induced activation of TRPM7 regulates cell proliferation, migration, and viability of human prostate cells. Biochim. Biophys. Acta.

[31] Maugeri M., Nawaz M., Papadimitriou A., Angerfors A., Camponeschi A., Na M., Holtta M., Skantze P., Johansson S., Sundqvist M. (2019). Linkage between endosomal escape of LNP-mRNA and loading into EVs for transport to other cells. Nat. Commun..

[32] Naseri N., Valizadeh H., Zakeri-Milani P. (2015). Solid Lipid Nanoparticles and Nanostructured Lipid Carriers: Structure, Preparation and Application. Adv. Pharm. Bull..

[33] Garcon N., Chomez P., Van Mechelen M. (2007). GlaxoSmithKline Adjuvant Systems in vaccines: Concepts, achievements and perspectives. Expert Rev. Vaccines.

[34] Thomann-Harwood L.J., Kaeuper P., Rossi N., Milona P., Herrmann B., McCullough K.C. (2013). Nanogel vaccines targeting dendritic cells: Contributions of the surface decoration and vaccine cargo on cell targeting and activation. J. Control. Release.

